# Health-related quality of life in early onset scoliosis patients treated with the spring distraction system: what to expect in the first 2 years after surgery

**DOI:** 10.1007/s43390-023-00777-9

**Published:** 2023-11-11

**Authors:** Justin V. C. Lemans, Anouk Top, Casper S. Tabeling, E. Pauline Scholten, Hilde W. Stempels, Tom P. C. Schlösser, René M. Castelein, Moyo C. Kruyt

**Affiliations:** 1 Department of Orthopaedic Surgery, University Medical Center Utrecht, P.O. Box 85500, 3508 GA Utrecht, The Netherlands; 2 Department of Developmental BioEngineering, Twente University, Enschede, The Netherlands

**Keywords:** Spring distraction system, Growing rods, Growth-friendly, Early-onset scoliosis, Quality of life, EOSQ-24

## Abstract

**Purpose:**

The Spring Distraction System (SDS) is a novel “growth-friendly” implant for the treatment of Early-Onset Scoliosis (EOS). This prospective study aims to determine the evolution of the “24-Item Early-Onset Scoliosis Questionnaire” (EOSQ-24) scores during 2-year follow-up after SDS surgery. Secondary aims include investigating the relation between EOSQ-24 scores and EOS etiology, and evaluating the impact of an unplanned return to the operating room (UPROR) on HRQoL.

**Methods:**

All SDS patients with at least 2-year follow-up were included. Caregivers completed the EOSQ-24 pre-operatively, post-operatively, and at 6, 12, and 24 month follow-up. Mean total and -domain scores were graphed over time. Repeated-measures ANOVA analyzed the influence of etiology on EOSQ-24 scores. Multiple regression analyzed associations between UPRORs and EOSQ-24 scores.

**Results:**

Forty-nine patients were included. Mean total EOSQ-24 scores decreased from 70 pre-operatively to 66 post-operatively, then gradually increased to 75 (24 months). Most domains exhibited changes over time, with initial declines, but eventually surpassing pre-operative levels after 2-year follow-up. Neuromuscular/Syndromic patients had lower scores, but showed similar improvements over time compared with other etiologies. Multiple regression showed lower Parental Burden domain score (− 14 points) in patients with UPRORs, although no significant reductions were found in total score, or in other domains.

**Conclusion:**

HRQoL decreases immediately following SDS surgery but quickly recovers and exceeds pre-operative levels at 2-year follow-up in all domains. Neuromuscular/Syndromic patients have lower initial scores, but progress similarly over time. UPRORs do not influence EOSQ-24 scores, except for a negative impact on the Parental Burden domain in the short term.

**Level of Evidence:**

III.

**Supplementary Information:**

The online version contains supplementary material available at 10.1007/s43390-023-00777-9.

## Introduction

**Fig. 1:**
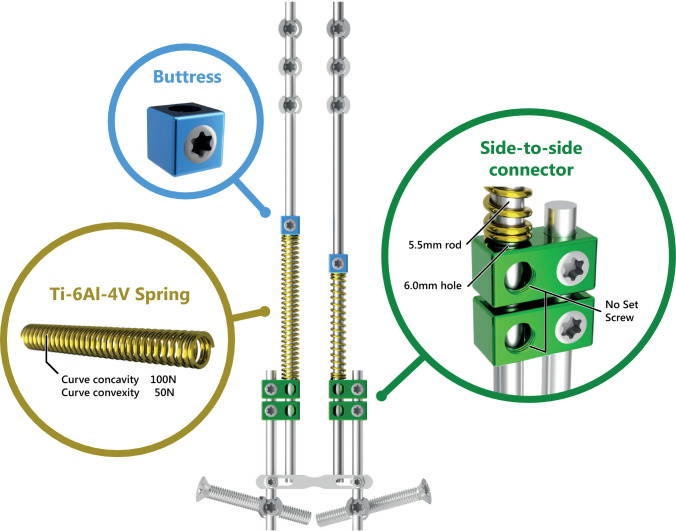
Spring distraction system concept. The SDS is a “growth-friendly” system that provides a continuous distraction force during follow-up, without the need for repeated lengthenings. Different configurations are used for different curve morphologies. The SDS consists of standard instrumentation to which several components are added. *Green:* Two side-to-side connectors that have an oversized hole that can accommodate a sliding CoCr rod. *Gold*: Uni- or bilateral Ti6Al4V springs that are mounted on the rod and which can be compressed. *Blue:* A buttress block that compresses the spring against the side-to-side connector

Early-onset scoliosis (EOS) is a deformity of the spine and trunk that occurs before the age of ten and can lead to substantial morbidity if left untreated [1]. For severe progressive curves, several surgical treatment options are available, including the traditional growing rod (TGR) and magnetically controlled growing rod (MCGR). While both systems offer adequate curve correction and growth, complication rates are high, and the repeated lengthenings are a burden for the patient, their caregivers, and the healthcare system [2, 3]. At our institution, the spring distraction system was developed out of an unmet patient need (Fig. 1). It provides continuous and dynamic distraction forces and achieves stable curve correction and near-normal growth without the need for repetitive surgical or outpatient lengthenings [4–7]. However, the effect of SDS treatment on health-related quality of life (HRQoL) has not yet been characterized.

To study HRQoL in EOS, the “24-Item Early-Onset Scoliosis Questionnaire” (EOSQ-24) was developed [8]. The EOSQ-24 is a questionnaire filled out by parents or caregivers and has been cross-culturally validated in several languages [9–11]. It consists of 24 questions that correspond to 12 domains: General Health, Pain/Discomfort, Pulmonary Function, Transfer, Physical Function, Daily Living, Fatigue/Energy Level, Emotion, Parental Burden, Financial Burden, Child Satisfaction, and Parental Satisfaction. The total score and the scores of subdomains range from 0 to 100 points, with a higher score denoting better outcomes.

Several clinical studies using the EOSQ-24 have been performed, which have shown that differences between patients are correlated with EOS etiology [8, 12, 13]. Other studies have compared HRQoL between MCGR and TGR treatment [14, 15]. While the EOSQ-24 has the ability to discriminate between subgroups of patients with different curve severities or treatment status, very little is known on the over-time changes in HRQoL in EOS patients after surgery, as longitudinal data is sparse [13]. The natural course of HRQoL for untreated EOS is even more unclear, although one study found severely decreased HRQoL in adult, untreated EOS patients with large curves [16].

The main aim of the current study was to determine EOSQ-24 score evolution over time in EOS patients treated with the SDS. Secondary aims were to show differences in EOSQ-24 scores between different etiologic groups, and to determine whether unplanned return to the operating room (UPROR) leads to reduced HRQoL scores.

## Methods

### Study design and ethical review

The current study uses the data of the ongoing prospective clinical trial that was initiated in 2016 after ethical approval by the institutional review board of UMC Utrecht (METC 16/276). All EOS patients who received the SDS are prospectively followed as part of the GRADS (Growing Rods with the Addition of a Distraction Spring) cohort. Caregivers were asked to complete the validated, Dutch version of the EOSQ-24 pre-operatively, immediately post-operatively and at each follow-up visit [9]. Patients with a pre-operative EOSQ-24, at least two post-operative EOSQ-24's and at least 2-year follow-up were included in the current analysis.

### Surgical treatment

At UMC Utrecht, SDS treatment is offered to EOS patients with an indication for “growth-friendly” treatment, except in patients with diseases that compromise soft tissue- or bone strength such as osteogenesis imperfecta or Marfan syndrome. The surgical technique for SDS has been described previously [5, 7, 17]. Anchors and rods are implanted comparable to TGR, but the rods are not fixated in side-to-side connectors, but are allowed to slide freely in the connector. In addition, helical springs of 50N, 75N, or 100N are mounted on the rods unilaterally or bilaterally. Following surgery, braces are not applied and there are no restrictions in load-bearing and (sport) activities.

### Data collection

EOSQ-24 data of each patient was collected at 5 time points: pre-operatively, immediately post-operatively, and at 6-, 12-, and 24-month follow-up. In addition, patient- and curve-related baseline characteristics were obtained. These included age at surgery, sex, curve magnitude, etiology, and pre-operative coronal Cobb angle. For each patient, the presence or the absence of UPRORs within 2-year follow-up was determined [6].

### Multiple imputation for missing data

We used multiple imputation with parcel summary scores (PSS) as an advanced statistical method to address individual missing items and entire missing questionnaires [18]. This method provides reliable results, outperforming complete case analysis and mean imputation if  > 10% of subjects have missing data [18, 19]. The method comprises several steps:

1. Missing items are identified at each time point.
2. PSS are created; these are the average of the available questions which are used as surrogate for the missing question itself. This information is used as a predictor to impute questions at other time points. The temporary score is only used in the imputation process.
3. Through the PSS, missing items are imputed based on a combination of other filled-out values in the same questionnaire, values of past and future completed questionnaires of the same patient, and patient etiology.
4. All imputed datasets are merged into one multiple imputation dataset. From the single question scores, category and total scores are calculated and analyzed.

### Statistical analyses

Following multiple imputation, mean EOSQ-24 scores were calculated and graphed over time. To test whether the total EOSQ-24 score or any of its domains changed over time, a mixed repeated-measures ANOVA was performed for each category, with time as the within-subjects factor and etiology as the between-subjects factor. Several post hoc analyses were performed: Pre-operative vs. post-operative, post-operative vs. 6 months, 6 months vs. 1 year, 1 vs. 2 years, pre-operative vs. 2 years, and post-operative vs. 2 years.

The effect of etiology was analyzed in the mixed repeated-measures ANOVA. Due to the small number of syndromic EOS patients, these patients were added to the neuromuscular group, as it has been previously shown that both groups have similar EOSQ-24 scores [12]. Similar to the time effect, we first determined whether there were significant differences in the F statistic followed by post hoc analyses: Idiopathic vs. Congenital, Idiopathic vs. Neuromuscular and Congenital vs. Neuromuscular. In addition, the interaction effect between time and etiology was evaluated to determine whether EOSQ-24 score evolution was different between etiologies.

To investigate the effect of UPRORs on EOSQ-24 score, we first investigated the difference in EOSQ-24 scores before and after UPRORs in all patients who suffered an UPROR with a paired t-test. As the trend over time in patients without UPRORs is unknown and could be subject to confounding, we also performed a multiple regression analysis in all patients with EOSQ-24 (domain) score as the dependent variable and the onset of an UPROR within the study period as the independent variable. For patients with UPRORs, the first EOSQ-24 after the UPROR was used as the dependent variable. For patients without UPRORs, we used the EOSQ-24 at 2-year follow-up. These different points were chosen to maximize potential differences and provide a worst-case scenario analysis. As we expected pre-operative EOSQ-24 values and etiology to be potential confounders, these were added in the model so that the effect of UPRORs on EOSQ-24 score could be independently investigated. We also repeated the analysis using the EOSQ-24 score at 2-year follow-up in all patients, to evaluate the effects of UPRORs on long-term HRQoL.

Statistical significance was set at *p* < 0.05. The false discovery rate (i.e., the rate of Type I errors due to multiple testing) for the post hoc analyses after analyzing the main effects was controlled at 5% through the Benjamini–Hochberg method, and the adjusted *p *values were calculated [7]. All statistical procedures were performed with IBM SPSS Statistics for Windows, Version 27.0. (IBM Corp., Armonk, NY, USA). Figures were created with GraphPad Prism version 9.3.0 (GraphPad Software, San Diego, CA, USA).

## Results

### Baseline demographics

Out of 59 SDS patients with 2-year follow-up, 49 were included for analysis. Ten patients did not have a filled-out pre-operative EOSQ-24 and were excluded. Mean age at surgery was 8.7 years, 49% of patients were girls (Table 1). Almost all neuromuscular patients (22/23; 96%) were non-ambulatory. All patients with other etiologies were ambulatory. The mean pre-operative coronal Cobb angle was 70°, which was corrected to 39° and which was mostly maintained at 2-year follow-up at 45°. Sixteen patients (33%) required UPRORs for surgical site infections, or implant-related complications.

**Table 1:** Baseline characteristics

Variable	
Age at surgery (SD)	8.7 years (2.0)
Gender (%)	
Female	24/49 (49%)
Male	25/49 (51%)
First growth implant vs. conversion (%)	
Primary	42/49 (86%)
Conversion	7/49 (14%)
Etiology (%)	
Congenital	12/49 (25%)
Idiopathic	11/49 (22%)
Neuromuscular	23/49 (47%)
Syndromic	3/49 (6%)
Ambulatory (%)	27/49 (55%)
Pre-operative Cobb angle (SD)	70.3° (23.1)
Patients with UPROR < 2 years	16/49 (33%)

### Missing data

During data collection, we observed that 25 patients (51%) had no missing data, while 7 patients (14%) filled out all questionnaires but had at least one missing answer. In 17 patients (35%), an entire questionnaire was missing during follow-up. In total, 8.0% of items (i.e., individual EOSQ-24 questions) were missing and had to be imputed (if a complete questionnaire had not been filled out, this amounted to 24 missing items). Missingness of data was observed to be related to etiology (i.e., missing at random), but was not related to the magnitude of EOSQ-24 score itself.

### EOSQ-24 scores over time

**Fig. 2:**
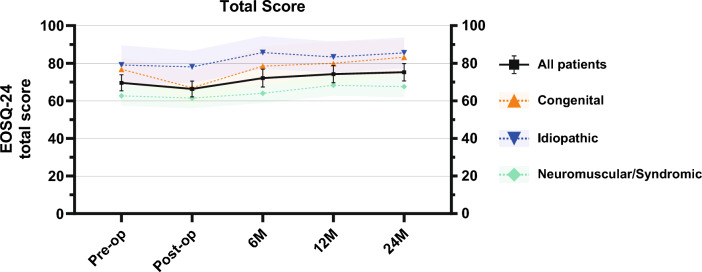

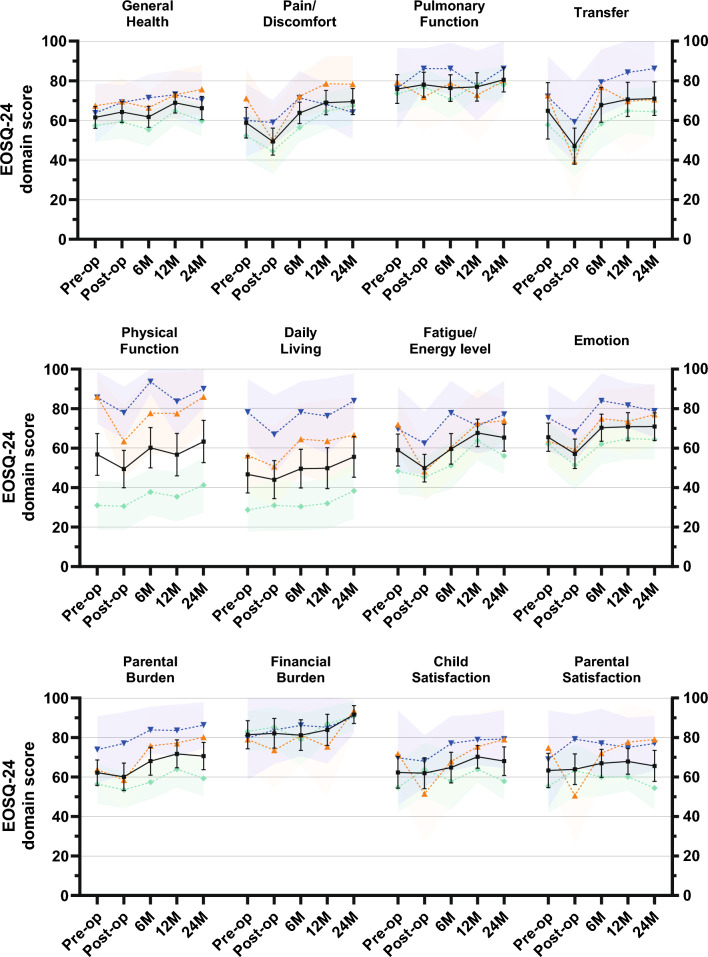
Health-related quality of life over time. Black line and whiskers indicate the mean and 95% confidence interval of the entire patient cohort. Colored dashed lines and shaded area indicate the mean and 95% confidence interval for each etiologic group

EOSQ-24 scores over time are shown in Table 2 and graphed in Fig. 2. Mean total EOSQ-24 score decreased from 70 ± 15 pre-operatively to 66 ± 15 post-operatively. Scores normalized at 6-month follow-up at 72 ± 17 and increased further to 74 ± 16 at 1-year and 75 ± 16 at 2-year follow-ups. Several domains did not show significant changes over time (General Health, Pulmonary Function, Financial Burden, Child Satisfaction and Parental Satisfaction). The results of post hoc analyses are shown in Table 3. In all domains, EOSQ-24 scores decreased immediately post-operatively, but recovered completely within 6 months and were higher at 2-year follow-up compared to pre-operatively. These changes were statistically significant for the Total Score (+ 5.6), Pain/Discomfort- (+ 11), Daily Living- (+ 8.9), and Parental Burden domains (+ 8.4).

**Table 2:** Results of mixed repeated-measures ANOVA: effect of time

	Pre-operative	Post-operative	6 months	12 months	24 months	*p* value^a^
Total	70 (SD 15)	66 (SD 15)	72 (SD 17)	74 (SD 16)	75 (SD 16)	< 0.001^b^
General Health	62 (SD 19)	64 (SD 18)	62 (SD 19)	69 (SD 18)	66 (SD 21)	0.135
Pain/Discomfort	59 (SD 27)	49 (SD 24)	64 (SD 19)	69 (SD 21)	70 (SD 23)	< 0.001
Pulmonary Function	76 (SD 25)	78 (SD 22)	76 (SD 23)	77 (SD 25)	81 (SD 21)	0.564^b^
Transfer	65 (SD 50)	47 (SD 32)	68 (SD 31)	71 (SD 30)	71 (SD 29)	< 0.001^b^
Physical Function	57 (SD 37)	49 (SD 33)	60 (SD 36)	57 (SD 38)	63 (SD 37)	0.006^b^
Daily Living	47 (SD 33)	44 (SD 34)	50 (SD 34)	50 (SD 36)	56 (SD 36)	0.022
Fatigue/Energy level	59 (SD 28)	50 (SD 24)	60 (SD 27)	68 (SD 24)	65 (SD 24)	< 0.001
Emotion	66 (SD 25)	57 (SD 26)	70 (SD 24)	71 (SD 25)	71 (SD 24)	0.007^b^
Parental Burden	62 (SD 23)	60 (SD 24)	68 (SD 25)	72 (SD 25)	71 (SD 24)	< 0.001^b^
Financial Burden	81 (SD 25)	82 (SD 26)	81 (SD 27)	84 (SD 27)	92 (SD 16)	0.097^b^
Child Satisfaction	62 (SD 28)	62 (SD 27)	65 (SD 27)	70 (SD 20)	68 (SD 25)	0.106^b^
Parental Satisfaction	63 (SD 30)	64 (SD 27)	67 (SD 25)	68 (SD 23)	66 (SD 27)	0.478^b^

^a^Based on time effect in mixed repeated-measures ANOVA

^b^Greenhouse–Geisser correction was used due to non-sphericity of data

**Table 3:** Post hoc analyses of different time points in the mixed repeated-measures ANOVA

	Pre-operative vs. post-operative		Post-operative vs. 6 months		6 months vs. 1 year		1 vs. 2 years		Pre-operative vs. 2 years		Post-operative vs. 2 years	
	Change^a^	*p* value^b^	Change^a^	*p* value^b^	Change^a^	*p* value^b^	Change^a^	*p* value^b^	Change^a^	*p* value^b^	Change^a^	*p* value^b^
Total	− 3.3	0.131	5.8	0.002	2.1	0.277	1.0	0.578	5.6	0.008	8.9	< 0.001
General Health	Main effect not statistically significant											
Pain/Discomfort	− 9.5	0.058	15	0.005	5.2	0.170	0.5	0.942	11	0.023	20	< 0.001
Pulmonary Function	Main effect not statistically significant											
Transfer	− 18	0.002	21	0.002	2.8	0.614	0.4	0.964	6.2	0.373	24	< 0.001
Physical Function	− 7.4	0.120	11	0.037	− 3.5	0.442	6.6	0.120	6.5	0.105	14	0.002
Daily Living	− 2.7	0.613	5.6	0.270	0.2	0.976	5.8	0.166	8.9	0.038	12	0.006
Fatigue/Energy level	− 9.1	0.079	9.7	0.037	8.1	0.105	− 2.4	0.578	6.3	0.166	15	< 0.001
Emotion	− 8.4	0.131	13	0.002	0.5	0.942	0.1	0.976	5.4	0.284	14	0.005
Parental Burden	− 2.1	0.607	7.9	0.033	3.7	0.225	− 1.1	0.745	8.4	0.037	11	0.002
Financial Burden	Main effect not statistically significant											
Child Satisfaction	Main effect not statistically significant											
Parental Satisfaction	Main effect not statistically significant											

^a^Positive value denotes an increase in HRQoL over time

^b^Benjamini–Hochberg adjusted *p* value with false discovery rate set at 0.05

### Influence of etiology on EOSQ-24 scores

The effect of etiology is seen in Table 4. EOSQ-24 scores across etiologies are shown in Fig. 2. There were significant differences in all domains except the Pain/Discomfort, Pulmonary Function, and Transfer and Financial Burden domains, with the lowest scores for neuromuscular patients, followed by congenital patients and the highest scores for idiopathic patients. The differences between idiopathic and neuromuscular patients ranged between 10 and 51 points, depending on domain. The differences between congenital and neuromuscular patients ranged between 8.6 and 43 points. The largest differences between etiologies were seen in Physical Function domain (mean score across time points; idiopathic: 86, congenital: 78, neuromuscular: 35) and the Daily Living domain (mean score across time points; idiopathic: 77, congenital: 60, neuromuscular: 32). Differences between idiopathic and congenital patients were relatively small (− 1.0 to 17) and not significant in any domain.

**Table 4:** Results of mixed repeated-measures ANOVA: effect of etiology

	Effect of etiology	Pairwise comparisons of etiology^a^						Interaction of time × etiology
		I—C		I—NM		C—NM		
	*p* value	Mean	*p* value^b^	Mean	*p* value^b^	Mean	*p* value^b^	*p* value
Total	< 0.001	5.3	0.344	18	0.001	12	0.016	0.229^c^
General Health	0.023	− 1.0	0.856	10	0.070	11	0.041	0.837
Pain/Discomfort	0.064	Main effect not statistically significant						0.224
Pulmonary Function	0.556	Main effect not statistically significant						0.409^c^
Transfer	0.051	Main effect not statistically significant						0.615^c^
Physical Function	< 0.001	8.1	0.452	51	< 0.001	43	< 0.001	0.418^c^
Daily Living	< 0.001	17	0.167	45	< 0.001	28	0.009	0.711
Fatigue/Energy level	0.019	6.5	0.462	19	0.027	12	0.116	0.109
Emotion	0.030	8.0	0.344	17	0.028	8.6	0.229	0.883^c^
Parental Burden	0.003	10	0.261	23	0.005	13	0.081	0.340^c^
Financial Burden	0.719	Main effect not statistically significant						0.691^c^
Child Satisfaction	0.033	5.5	0.462	15	0.036	9.6	0.163	0.110^c^
Parental Satisfaction	0.015	4.6	0.542	17	0.027	12	0.081	0.075^c^

^a^A positive value denotes that the first group has higher scores compared to the second group and vice versa

^b^Benjamini–Hochberg adjusted *p* value with false discovery rate set at 0.05

^c^Greenhouse–Geisser correction was used due to non-sphericity of data

Interactions between time and etiology were not seen in any domain, suggesting that the improvement in EOSQ-24 scores over time was similar in all etiological groups.

### Influence of complications on EOSQ-24 scores

The pre- and post-UPROR EOSQ-24 scores in the 16 patients with UPRORs can be seen in Table 5. In patients with UPRORs, the mean time between the UPROR and the next EOSQ-24 was 94 ± 50 days. In many domains, an improvement was seen in the post-UPROR EOSQ-24 scores. Although not statistically significant, this coincides with the trend of improvement of HRQoL over time seen in all patients. The results of multiple regression investigating the effect of UPRORs in all patients are shown in Table 6. A significant decrease in EOSQ-24 score was seen in the Parental Burden domain (− 14 points, 95% CI − 26; − 1.6) in patients with an UPROR. In addition, the presence of UPRORs was correlated with sizable decreases that approached significance in the Transfer (− 11 points, 95% CI − 30; 8.0), Child Satisfaction (-7.6 points, 95% CI − 25; 9.3), and Parental Satisfaction (− 11 points, 95% CI − 25; 3.9) domains. The results of multiple regression with 2-year EOSQ-24 values in all patients are shown in Supplement 1. When looking at the long-term follow-up, the (modest) negative effect of UPRORs on HRQoL disappears, except for the Satisfaction domains, where a trend toward further decrease is seen in the long term.

**Table 5:** Comparison of EOSQ-24 scores before and after an UPROR

	Score before UPROR	Score after UPROR	Paired difference (95% CI)^a^	*p* value
Total	66	70	4.0 (− 0.82; 8.8)	0.103
General Health	63	62	− 0.94 (− 14; 12)	0.884
Pain/Discomfort	53	63	10 (− 5.0; 25)	0.193
Pulmonary Function	75	79	3.8 (− 8.0; 16)	0.532
Transfer	60	58	− 1.7 (− 29; 25)	0.893
Physical Function	45	55	9.4 (− 7.1; 26)	0.264
Daily Living	42	55	13 (− 5.3; 32)	0.159
Fatigue/Energy level	54	61	7.0 (− 7.4; 21)	0.338
Emotion	59	69	10 (− 5.3; 25)	0.194
Parental Burden	57	59	2.7 (− 5.4; 11)	0.517
Financial Burden	83	87	3.9 (− 16; 23)	0.692
Child Satisfaction	68	61	− 6.6 (− 23; 10)	0.437
Parental Satisfaction	59	57	− 2.3 (− 17; 13)	0.753

Analyzed in all patients with an UPROR within 2 years (*N* = 16)

^a^ A positive number denotes higher EOSQ-24 score after the UPROR compared to before the UPROR

**Table 6:** Multiple regression analysis investigating the effect of UPRORs on HRQoL

	Proportion of explained variation	Constant	Pre-operative domain score	Presence of UPROR	Etiology	
					Congenital	Neuromuscular
	*R*^2^ value	*B* (95% CI)	*B* (95% CI)	*B* (95% CI)	*B* (95% CI)	*B* (95% CI)
Total	0.52	41 (18; 63)	0.56 (0.28; 0.84)	− 3.7 (− 11; 3.6)	0.31 (− 8.9; 9.5)	− 8.9 (− 18; 0.53)
General Health	0.37	38 (12; 64)	0.54 (0.20; 0.88)	− 4.1 (− 16; 8.2)	2.8 (− 11; 16)	− 7.5 (− 20; 5.4)
Pain/Discomfort	0.26	47 (28; 65)	0.33 (0.08; 0.57)	− 4.3 (− 17; 8.1)	9.7 (− 6.3; 26)	− 1.6 (− 16; 13)
Pulmonary Function	0.25	59 (37; 81)	0.35 (0.11; 0.59)	0.55 (− 13; 14)	− 7.3 (− 23; 8.2)	− 12 (− 26; 1.9)
Transfer	0.15	67 (42; 92)	0.19 (− 0.09; 0.48)	− 11 (− 30; 8.0)	− 7.4 (− 28; 14)	− 13 (− 32; 6.7)
Physical Function	0.68	32 (5.3; 59)	0.70 (0.40; 1.0)	− 1.2 (− 15; 12)	− 6.1 (− 24; 12)	− 15 (− 37; 7.9)
Daily Living	0.55	35 (7.8; 62)	0.63 (0.29; 1.0)	9.2 (− 6.8; 25)	− 6.1 (− 26; 14)	− 21 (− 44; 1.1)
Fatigue/Energy level	0.41	42 (22; 62)	0.48 (0.24; 0.71)	1.4 (− 12; 15)	− 2.8 (− 19; 14)	− 14 (− 30; 2.6)
Emotion	0.17	56 (29; 83)	0.27 (− 0.05; 0.59)	0.7 (− 14; 16)	3.9 (− 14; 22)	− 11 (− 27; 5.7)
Parental Burden	0.47	46 (22; 69)	0.51 (0.21; 0.81)	− 14 (− 26; − 1.6)	5.6 (− 9.1; 20.4)	− 11 (− 25; 2.6)
Financial Burden	0.08	76 (54; 99)	0.18 (− 0.07; 0.43)	− 3.5 (− 16; 8.9)	4.3 (− 11; 19)	− 3.1 (− 17; 11)
Child Satisfaction	0.18	73 (51; 94)	0.09 (− 0.18; 0.34)	− 7.6 (− 25; 9.3)	2.2 (− 16; 20)	− 15 (− 32; 1.6)
Parental Satisfaction	0.27	63 (43; 83)	0.22 (− 0.01; 0.45)	− 11 (− 25; 3.9)	2.9 (− 15; 21)	− 15 (− 31; 1.6)

Analyzed with data from all patients (*N* = 49). For patients with an UPROR, the EOSQ-24 result at the first time point after the UPROR was used as the dependent variable. For patients without an UPROR, the 2-year follow-up EOSQ-24 was used as the dependent variable

## Discussion

The present study investigated changes in HRQoL over time in a diverse EOS population that underwent SDS treatment. We observed a general trend where EOSQ-24 scores declined immediately following surgery, but recovered to baseline or higher within 6 months. At 2-year follow-up, the mean score in each domain was higher than pre-operative levels, with a statistically significant relation for the total score, and the Pain/Discomfort, Daily Living, and Parental Burden domains. These changes are considered clinically relevant as they surpass the minimal clinically important difference (MCID) when using distribution-based approaches (e.g., a change of > 1 SEM or > 0.2 SD) [20]. However, formal MCIDs for the EOSQ-24 have not yet been established, which makes conclusions regarding clinical relevance preliminary. However, not all EOSQ-24 domain scores improved over time, such as the Parental Satisfaction domain.

The findings from this study can be utilized to counsel patients and their caregivers about the expected subjective health changes during the initial two years after surgery. It is promising to note that HRQoL continued to improve with longer follow-up in all patient groups, possibly due to the SDS allowing patients to resume normal activities without restrictions and periodic lengthenings. However, it remains to be seen if this positive trend continues until after SDS graduation.

Neuromuscular/Syndromic patients showed lower initial scores in several domains as expected, but the improvement over time was similar to their peers with other etiologies. Interestingly, no correlation was seen between etiology and the Pulmonary Function domain, while one would expect lower scores in neuromuscular patients. This observation aligns with a previous study indicating that the Pulmonary Function domain of the EOSQ-24 demonstrates high variability and limited association with pulmonary function test (PFT) results, particularly in patients with a forced vital capacity < 40% [21]. We do not routinely perform PFTs, as these are burdensome and oftentimes cannot be reliably obtained in young patients, especially if there is developmental delay [22].

Previous studies have investigated EOSQ-24 scores in different settings. Ramo et al. showed in a large cross-sectional study of over 600 EOS patients that neuromuscular and syndromic patients have significantly lower EOSQ-24 scores compared to idiopathic and congenital patients, and that the latter two groups have very similar scores [12]. In a follow-up study, Shaw et al. showed that “growth-friendly” treatment mainly stabilizes HRQoL after 2 years, and that the evolution is similar in different etiologies, which is in line with our own data [23]. However, in contrast to our study, they identified no domains where patients improved at 2-year follow-up compared to baseline. However, the MCID used in that study (> 20% increase) could be considered conservative.

In patients with UPRORS, no decline in HRQoL was observed over time. Instead, a trend of improvement was seen, aligning with the general trend in the entire patient group. After adjusting for curve etiology and pre-operative score in the linear regression, we found significantly lower scores in the Parental Burden domain (− 14 points, 95% CI − 26; − 1.6). There was also a trend towards worse scores in the Transfer (− 11 points, 95% CI − 30; 8.0) and Parental Satisfaction (− 11 points, 95% CI − 25; 3.9) domains, but no decrease in the total score. This contrasts with a previous study, which saw a decrease in total EOSQ-24 score [24]. This could be due to several reasons. First, the mean time between UPRORs and the next questionnaire was > 90 days. In that timeframe, EOSQ-24 scores could have decreased, but normalized to baseline again. Second, it is possible that the current analysis was underpowered to detect the relatively small differences between groups. Third, UPRORs are generally less extensive surgeries compared to the primary surgery. This may result in a smaller decrease in scores compared to the primary surgery, which the EOSQ-24 may not be able to detect. When looking at the long-term follow-up in all patients, no relation was seen between UPRORs and HRQoL, suggesting that UPRORs do not negatively affect HRQoL in the long-term. We chose UPRORs instead of complications as they could more objectively be defined as being present compared to complications.

A major strength of the current study is its longitudinal design. The current study is the first to investigate the change in HRQoL across many fixed post-operative time points in a prospective EOS patient cohort, where other studies often opt for a cross-sectional design [14, 15]. Additionally, we utilized multiple imputation with PSS to account for missing EOSQ-24 item values, enabling advanced statistical analysis and increasing study power compared to a complete case analysis, which would exclude nearly 50% of participants. Limitations of the study include the relatively low sample size per condition and the short follow-up of 2 years. However, the included SDS patients will be followed up with the EOSQ-24 until after skeletal maturity, which may provide information for the long-term follow-up of HRQoL. Another limitation is the absence of a comparison group, making it challenging to determine whether patients undergoing SDS treatment have different HRQoL compared to those treated with another implant, or those who have not been treated surgically at all.

## Conclusion

Following SDS surgery, EOSQ-24 scores decrease post-operatively in several domains. However, all scores recover to pre-operative levels within 6 months, and in several domains, scores exceed pre-operative levels at 2-year follow-up. Patients with neuromuscular/syndromic EOS etiology initially score lower in several domains, but their progression following surgery is similar to the other etiologies. The total EOSQ-24 score and the score in most EOSQ-24 domains are unaffected by the presence of UPRORs although they lead to worse scores in the Parental Burden domain in the short term.

### Supplementary Information

Below is the link to the electronic supplementary material.

Supplementary file1 (DOCX 18 KB)

## Data Availability

The data that support the findings of this study are available from the corresponding author upon reasonable request.
