# Supplementary material for: Health-related quality of life in early onset scoliosis patients treated with the spring distraction system: what to expect in the first 2 years after surgery
Source: Spine Deform. 2023 Nov 11;12(2):489–99. doi: 10.1007/s43390-023-00777-9 (PMC10867097; doi:10.1007/s43390-023-00777-9)
Supplement: Supplementary file 1 — Supplementary file1 (DOCX 18 KB) [file 43390_2023_777_MOESM1_ESM.docx]

**Supplement 1: Multiple regression analysis investigating the effect of UPRORs on HRQoL**

|  | **Proportion of explained variation** | **Constant** | **Pre-operative domain score** | **Presence of UPROR** | **Etiology** | |
| --- | --- | --- | --- | --- | --- | --- |
|  |  |  |  |  | **Congenital** | **Neuromuscular** |
|  | **R^2^ value** | **B (95% CI)** | **B (95% CI)** | **B (95% CI)** | **B (95% CI)** | **B (95% CI)** |
| **Total** | 0.50 | 39 (15; 62) | 0.59 (0.32; 0.87) | -1.4 (-8.8; 6.0) | -0.89 (-11; 9.0) | -7.9 (-18; 2.1) |
| **General Health** | 0.40 | 35 (12; 59) | 0.56 (0.28; 0.85) | -6.3 (-17; 4.0) | 4.0 (-10; 18) | -5.3 (-18; 7.1) |
| **Pain/Discomfort** | 0.25 | 40 (20; 61) | 0.38 (0.14; 0.63) | 4.5 (-8.4; 17) | 9.9 (-7.8; 28) | 5.3 (-11; 21) |
| **Pulmonary Function** | 0.29 | 53 (32; 75) | 0.42 (0.19; 0.66) | 4.5 (-7.2; 16) | -7.8 (-23; 7.7) | -8.2 (-22; 5.7) |
| **Transfer** | 0.11 | 77 (49; 105) | 0.13 (-0.16; 0.43) | -1.5 (-20; 17) | -16 (-39; 8.2) | -20 (-41; 2.2) |
| **Physical Function** | 0.68 | 21 (-6.5; 48) | 0.81 (0.53; 1.1) | -2.0 (-16; 12) | -4.2 (-23; 14) | -3.9 (-26; 18) |
| **Daily Living** | 0.58 | 24 (-5.8; 55) | 0.74 (0.42; 1.1) | 7.2 (-8.4; 23) | -1.4 (-23; 21) | -10 (-36; 16) |
| **Fatigue/Energy Level** | 0.38 | 48 (28; 68) | 0.41 (0.19; 0.63) | 5.9 (-6.3; 18) | -4.6 (-21; 12) | -14 (-30; 2.1) |
| **Emotion** | 0.16 | 59 (32; 86) | 0.26 (-0.05; 0.56) | 4.4 (-10; 19) | 0.87 (-19; 20) | -12 (-29; 5.3) |
| **Parental Burden** | 0.46 | 50 (27; 74) | 0.51 (0.23; 0.79) | -8.0 (-20; 3.9) | -0.42 (-16; 15) | -16 (-31; -1.6) |
| **Financial Burden** | 0.12 | 75 (59; 93) | 0.19 (0.00; 0.38) | 3.3 (-6.7; 13) | 2.8 (-10; 16) | -1.3 (-13; 11) |
| **Child Satisfaction** | 0.23 | 76 (53; 99) | 0.08 (-0.17; 0.33) | -10 (-25; 4.3) | 0.27 (-19; 19) | -18 (-35; -0.40) |
| **Parental Satisfaction** | 0.31 | 64 (43; 86) | 0.23 (-0.01; 0.46) | -14 (-29; 0.9) | 1.5 (-18; 21) | -16 (-34; 1.1) |
| Analyzed with data from all patients (N=49). For all patients, the 2-year follow-up EOSQ-24 was used as the dependent variable. | | | | | | |
